# Uncovering drug repurposing candidates for head and neck cancers: insights from systematic pharmacogenomics data analysis

**DOI:** 10.1038/s41598-021-03418-1

**Published:** 2021-12-14

**Authors:** Annie Wai Yeeng Chai, Aik Choon Tan, Sok Ching Cheong

**Affiliations:** 1 Translational Cancer Biology Research Unit, Cancer Research Malaysia, Cancer Research Malaysia, 2nd Floor Outpatient Center, Subang Jaya Medical Center, No. 1, Jalan SS12/1A, 47500 Subang Jaya, Selangor Malaysia; 2 Department of Biostatistics and Bioinformatics, Moffitt Cancer Center, Tampa, FL USA; 3 Department of Oral & Maxillofacial Clinical Sciences, Faculty of Dentistry, University of Malaya, Kuala Lumpur, Malaysia

**Keywords:** Cancer genetics, Head and neck cancer, Oral cancer, Tumour biomarkers

## Abstract

Effective treatment options for head and neck squamous cell carcinoma (HNSCC) are currently lacking. We exploited the drug response and genomic data of the 28 HNSCC cell lines, screened with 4,518 compounds, from the PRISM repurposing dataset to uncover repurposing drug candidates for HNSCC. A total of 886 active compounds, comprising of 418 targeted cancer, 404 non-oncology, and 64 chemotherapy compounds were identified for HNSCC. Top classes of mechanism of action amongst targeted cancer compounds included PI3K/AKT/MTOR, EGFR, and HDAC inhibitors. We have shortlisted 36 compounds with enriched killing activities for repurposing in HNSCC. The integrative analysis confirmed that the average expression of EGFR ligands (*AREG, EREG, HBEGF, TGFA,* and *EPGN*) is associated with osimertinib sensitivity. Novel putative biomarkers of response including those involved in immune signalling and cell cycle were found to be associated with sensitivity and resistance to MEK inhibitors respectively. We have also developed an RShiny webpage facilitating interactive visualization to fuel further hypothesis generation for drug repurposing in HNSCC. Our study provides a rich reference database of HNSCC drug sensitivity profiles, affording an opportunity to explore potential biomarkers of response in prioritized drug candidates. Our approach could also reveal insights for drug repurposing in other cancers.

## Introduction

Head and neck squamous cell carcinoma (HNSCC) is a deadly disease affecting more than 700,000 people worldwide^1^, unfortunately, effective treatment options are still lacking. Since 2006, the EGFR-targeting cetuximab remained the only approved molecular-targeted drug for HNSCC. In more recent years, two anti-PD1 immunotherapy drugs (pembrolizumab and nivolumab) have been approved for the treatment of recurrent and metastatic HNSCC. However, despite these advancements, only a minority of patients respond to these drugs, underscoring the unmet need to expand the treatment options for HNSCC.

High-throughput drug screening on cancer cell lines has been the starting point behind important drug discoveries and affords the opportunity to explore drug repurposing systematically. Some of these earlier large-scale efforts include the Cancer Cell Line Encyclopedia (CCLE)^2^, the Cancer Therapeutics Response Portal (CTRP)^3^ and the Genomics of Drug Sensitivity in Cancer (GDSC)^4,5^. More recently, a highly scalable method of pooled screening of a mixture of barcoded cell lines, called PRISM, an acronym for “profiling relative inhibition simultaneously in mixtures”^6^ presents a rich resource for drug repurposing opportunities that includes non-oncology drugs^7^. Amongst the cancer cell lines included in this resource, 28 human papilloma virus (HPV)-negative HNSCC cell lines were tested with 4,518 compounds, making this the largest dataset of drug response data for HNSCC. Herein, we endeavour to conduct a systematic in silico analysis of this PRISM repurposing dataset, focusing specifically on HPV-negative HNSCC, which is known to have poorer prognosis compared to HPV-positive HNSCC^8^. We aim to uncover the top mechanisms of action (MOA) in the compounds that show active killing in HNSCC and to identify promising compounds with biomarkers for HNSCC using computational approaches. Through this study, we also provide a readily accessible, and exploitable drug response reference dataset for HNSCC for the research community. Notably, our analysis helped to eliminate non-active compounds, revealing those that are most efficacious for HNSCC from the PRISM dataset. Further, we provide insights on the MOA landscape of active compounds, enabling the focus on downstream validation of these compounds for the treatment of HNSCC. We also demonstrated the utility of exploiting this pharmacogenomic dataset by providing examples of potential drug candidates, such as osimertinib and trametinib, along with markers of drug response, and suggestions of drug combinations to overcome drug resistance. Despite utilizing a publicly available dataset, cancer-specific analysis represent a valuable resource that will offer meaningful insights in uncovering unique drug candidates and pharmacogenomic biomarkers, which can otherwise be masked and remain unidentified in the large pan-cancer dataset.

## Materials and methods

### Processing of the PRISM drug repurposing dataset

The full PRISM primary screen dataset was downloaded from the Broad Institute Cancer Dependency Map (DepMap) portal (https://depmap.org/repurposing/-“primary_replicate_collapsed_logfold_change.csv”, 19Q4). The identities of the cell lines were mapped using the “primary-screen-cell-line-info.csv” file, while the information of the compounds tested were mapped using the “2- Compounds tested” tab from “Corsello_supplemental_tables.xlsx”. A total of 4,686 sets of log fold change values (logFC) [relative to dimethyl sulfoxide (DMSO)] was available for 4,518 compounds. Duplicated data for 161 compounds and triplicated data for three compounds (doxycycline, cefoselis and tempol) were averaged accordingly, generating a final matrix of 4,518 unique compounds on 28 HNSCC cell lines [Table S1]. Annotations of the compound name, drug category and MOA were also included in this matrix.

### Defining “sensitive” and “resistant” cell lines

The PRISM primary screen applied a fixed drug concentration of 2.5 µM and used the logFC relative to DMSO control as a measure of drug response^7^. To define “sensitive” vs “resistant”, we investigated the logFC datasets of FDA-approved drugs with a proven mutational biomarker of response to determine a rational threshold. Vemurafenib is used for the treatment of melanoma patients with BRAF V600E mutation. We compared the ln IC50 (half maximal inhibitory concentration) data from the GDSC on overlapping melanoma cell lines with the logFC to determine a suitable cutoff for logFC [Fig. S1A]. At the threshold of logFC = − 1, the specificity of 100% was achieved, with sensitivity at 80% and Youden’s index of 0.80, the maximum possible value we could get here. At this threshold, based on logFC data of PRISM, the specificity and sensitivity of 89% and 66% respectively were achieved [Fig. S1B]. Another drug gefitinib which is approved as first-line treatment for metastatic non-small cell lung cancer (NSCLC) patients with exon 19 deletions in EGFR was also investigated. At the threshold of logFC = − 1, the specificity and sensitivity were high at 88% and 75%, respectively [Fig. S1C]. Therefore, a cutoff value of logFC = − 1 was used to define active cell killing by the compounds, where cell lines with less than − 1 logFC were defined as being sensitive to the drug. Compounds with at least three sensitive HNSCC cell lines (above 10% of cell lines) were defined as “active” compounds, classification for each compound can be found in column “AH” of Table S1.

### Hierarchical clustering and heatmap visualization of the data matrix of active compounds using Morpheus

A data matrix of 418 active targeted cancer compounds, 404 active non-oncology compounds and 64 active chemotherapy compounds for the 28 HNSCC cell lines were uploaded onto the Morpheus tool, an online platform for data analysis and visualization (https://software.broadinstitute.org/morpheus/). To determine if there is any pattern of drug sensitivity that is associated with the subsite of the HNSCC, annotations for cancer subtype (oral squamous cell carcinoma (OSCC) or non-OSCC) were included. Further, the MOA was annotated for each compound. Hierarchical clustering analysis was performed on Morpheus, using the metric of “one minus spearman correlation” and “complete” linkage method, on both rows (compounds) and columns (cell lines). Interactive heatmaps can be accessed and explored by uploading the json files [Files S1–S3], onto the Morpheus tool [Fig. S3A]. These files are downloadable from figshare-https://doi.org/10.6084/m9.figshare.16443759.v1.

### t-distributed stochastic neighbour embedding (tsne) analysis and interactive visualization on RShiny app

The data matrix of 886 active compounds in 28 HNSCC cell lines consist of 24,808 data points, and the Morpheus tool was utilized to perform t-distributed stochastic neighbour embedding (tsne) analysis [Metric: one minus spearman rank correlation; Run on: Rows (compounds); Epsilon (learning rate): 100, perplexity: 30]. Other combinations of the epsilon and perplexity were also tested (300/30, 500/30), where compounds including EGFR inhibitors, MEK inhibitors (MEKi) were shown to cluster together^7^, were also recapitulated in our analysis. Representative T1 and T2 values from one of the ten runs were used for tsne plotting. Non-oncology compounds that consistently fall into proximity with (or consistently clustered close-by) the groups of targeted cancer compounds in all ten runs of tsne analysis were highlighted. To enable interactive visualization, we have created a RShiny app webpage: https://anniechai.shinyapps.io/tsne/, where details on each of the compounds can be obtained (Fig. 2D). Further, searches based on compounds or MOA or targets of interest can be conducted where results of the search query would be highlighted as enlarged dot(s) in the tsne plot as shown in Fig. S3B.

### Identification of compounds with preferential activity in HNSCC

The “Data explorer” tool from DepMap was utilized to perform the “two-class comparison” analysis (https://depmap.org/portal/interactive/), to compare the means of drug sensitivity data between two groups and generate the estimates of effect size and corresponding p-value. The “drug sensitivity (PRISM repurposing Primary screen 19Q4)” was selected as input dataset, with the 28 HNSCC cell lines being placed in the “in” group cell lines, while all other cell lines tested in PRISM were used as “out” group cell lines. A total of 179 unique compounds (ruxolitinib and calcitriol had duplicated screen results) were found to have preferential activity in HNSCC (*P* < 0.01 or − log10 (*P* value > 2) [Table S2]. Analysis was repeated with the input dataset of “drug sensitivity (PRISM repurposing Secondary screen (AUC) 19Q4)”. A total of 89 unique compounds showed statistically different means of drug sensitivity measurements between the HNSCC and the non-HNSCC cell lines [Table S3]. The 36 compounds commonly found using the primary and secondary screen data as input were annotated with their drug development status [Table S4].

### Gene set enrichment analysis (GSEA) for osimertinib-sensitive and -resistant HNSCC

To identify gene expression signatures and pathways that are associated with drug response, gene expression data for the HNSCC cell lines were extracted from the DepMap portal, Public 19Q4 release-“CCLE_expression.csv”^9,10^. This file consists of the RNA-sequencing-derived TPM gene expression data, in the form of Log2 transformed, and a pseudo-count of 1 − log2(TPM + 1). GCT file with gene expression data for the four most osimertinib-resistant cell lines (logFC > 0) [YD8, SNU46, SNU899, BICR6] and four most osimertinib-sensitive cell lines [YD38, SNU1076, SCC25, HSC3] were used as input file for gene set enrichment analysis (GSEA) using GenePattern^11^. Gene set database “c2.cp.kegg.v7.3.symbols.gmt [curated]” was selected. Full results of the GSEA comparing osimertinib-sensitive and -resistant HNSCC can be found in Table S5.

### Analysis workflow in identifying potential gene expression-based biomarkers for MEKi

The logFC data for all 20 MEKi were visualized using a heatmap in Morpheus [File S4, downloadable from figshare-https://doi.org/10.6084/m9.figshare.16443759.v1]. Hierarchical clustering was performed using a metric of “one minus spearman correlation” and “complete” linkage method. A distinct cluster of MEKi-resistant cell lines was observed, which included six lines: FADU, SNU1041, BICR18, HSC2, SNU46 and YD8. For the compounds, a few sub-branches consisting of eight active MEKi were found to share largely similar drug sensitivity profiles. This cluster consists of the three MEKi that were differentially enriched for killing activity in HNSCC (trametinib, AZD8330 and cobimetinib), as well as another five compounds (MEK162, Ro-4987655, AS-703026, PD-0325901 and TAK-733). The clinical indication and drug development status of these eight MEKi were manually curated [Table S6].

Differentially expressed genes (DEGs) were identified using the limma package (Bioconductor), by comparing the six resistant lines versus the six most sensitive lines (BICR6, CAL27, SCC25, PECAPJ49, SNU1076, YD38), based on their average logFC against the eight MEKi. The list of DEGs is available from Table S7. The 136 significantly upregulated DEGs in MEKi-sensitive lines were used as input query into the STRING portal (https://string-db.org, Version 11) network. Pathway enrichment analysis was performed using the portal and the most enriched REACTOME pathway-“Cytokines signalling in immune system” was highlighted in red (n = 24). The gene expressions of these 24 cytokines were used to correlate with MEKi average logFC, those with significant Pearson’s correlation (*P*-value < 0.05) were shortlisted as potential biomarkers of response to MEKi.

### GSEA comparison across multiple datasets

GCT file with gene expression matrix of the six MEKi-sensitive and six MEKi-resistant HNSCC was used as input for GSEA using GenePattern^11^. Gene set database “h.all.v7.4.symbol.gmt [Hallmarks]” was selected, with the rest following default parameters. Similar GSEA was also performed using the two following datasets as described.

Among 26 HNSCC lines tested with MEKi (trametinib) in GDSC, we identified the six most sensitive HNSCC (DOK, SAS, JHU-022, BB49-HNC, HO-1-N-1, SAT; mean IC50–0.18 µM) and six most resistant HNSCC (LB771-HNC, BICR10, OSC-20, HSC-2, SCC-4, HSC-4; mean IC50–4.62 µM). The gene expression dataset (RMA normalized) for these 12 cell lines were downloaded from the GDSC data portal (https://www.cancerrxgene.org/gdsc1000/GDSC1000_WebResources/Home.html).

For the drug screen by Lepikhova et al.^12^, six HNSCC lines were contained in each MEKi low and high sensitivity groups. High MEKi sensitivity group–UT_SCC_47, UT_SCC_42A, UT_SCC_106A, UT_SCC_40, UT_SCC_24A; Low MEKi sensitivity group-UT_SCC_54a, UT_SCC_6A, UT_SCC_21, UT_SCC_29, UT_SCC_28, UT_SCC_44. The microarray gene expression dataset of these 12 cell lines was downloaded from Gene Expression Omnibus (GEO) with the accession number GSE108062. GSEA was performed as described above, with Illumina HumanHT-12 V4.0 expression bead chip selected as the chip platform.

Hallmark gene sets enriched in MEKi-resistant groups of respective datasets (false discovery rate < 25%) were compared for similarity. Hallmarks commonly identified from all three datasets were highlighted in the Venn diagram. Full results of the GSEA are available in Table S8.

### Statistical analysis and Graph Pad Prism

The differences in the mean of logFC between the two groups were evaluated for their statistical difference using the two-tailed, unpaired Mann–Whitney test in the GraphPad Prism software 8.0.2. Pearson correlation between gene expression and drug sensitivity was also computed using GraphPad Prism, *P*-value < 0.05 is considered as statistically significant.

## Results

### Systematic analysis of high-throughput drug screen data on the HNSCC subset

**Figure 1:**
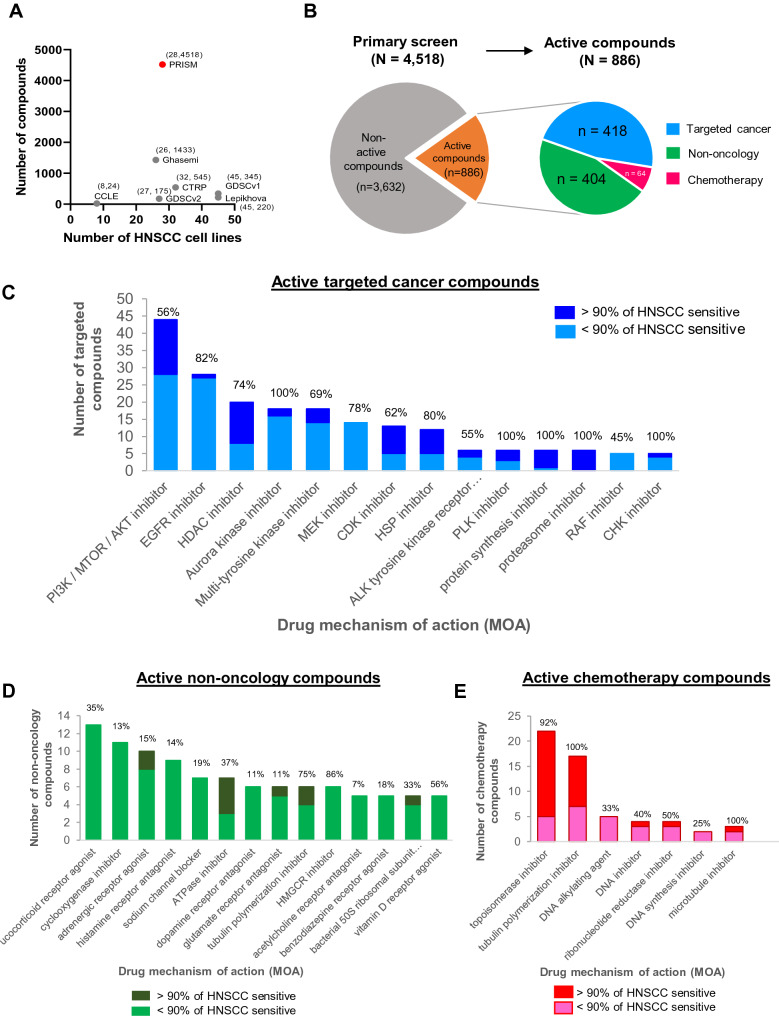
Analysis of large-scale drug screening conducted on HNSCC cell lines reveals drug mechanism of action (MOA) of active compounds. (**A**) Scale of high-throughput drug screening conducted on HNSCC cell lines. The recent PRISM repurposing dataset from the Broad Institute (Corsello et al. 2020) contains the largest number of compounds screened (n = 4,518), including non-oncology compounds (n = 3,466, 77%), that are not covered by other studies. Each dataset is annotated with the number of HNSCC cell lines, x and number of drugs screened, y-(x, y). (**B**) Using a pre-defined cutoff of -1 log fold change to identify sensitive cell lines to a particular drug, we identified 886 active compounds which have at least three sensitive HNSCC cell lines (> 10%). Among these 886 compounds, 418 were targeted cancer drugs, 404 non-oncology drugs and 64 were chemotherapy drugs. (**C**) The landscape of the active targeted cancer compounds, grouped according to their mechanisms of action (MOA), displaying only the top MOAs with at least five compounds. (**D**) The landscape of the top MOAs of active non-oncology compounds, displaying only the top MOAs with at least three compounds (**E**) The landscape of the top MOAs of active chemotherapy compounds, displaying only the top MOAs with at least five active compounds. For (**C**)–(**E**), the percentages indicated on each bar refer to the % of compounds defined as active over the total number of compounds tested on HNSCC lines for the respective MOAs. Different shades of colour depicting > or < 90% of sensitive cell lines for each MOA indicates drug selectivity where a large proportion of drugs with > 90% HNSCC sensitivity shows that drugs with that particular MOA is less selective.

Several high-throughput drug screens had include HNSCC cell lines, including the CCLE^2^, CTRP^13^, GDSC^4,14^ and PRISM^7^. Additionally, other independent research groups have also published their HNSCC screen outcomes, including Ghasemi et al.^15^ and Lephikova et al.^12^. These drug screens vary diversely in terms of screening techniques, platforms, cell lines and compounds. Amongst these, the “PRISM Repurposing dataset” conducted by the Broad Institute^7^, represents the largest resource for drug repurposing, and is the only dataset that includes non-oncology compounds published to date [Fig. 1A]. The primary screen of PRISM tested 4,518 compounds, including targeted cancer compounds (n = 956, 21%), chemotherapy (n = 96, 2%) and a vast variety of non-oncology compounds (n = 3,466, 77%). About 20% (n = 886) showed killing activity in at least three HNSCC lines (~ 10% of total 28), herein described as “active compounds” for HNSCC [Fig. 1B]. Here, we report the results from our systematic analysis of the PRISM dataset to uncover drug repurposing candidates for HNSCC.

### Landscape of the mechanisms of action of active compounds

Among the 886 active compounds, 418 were targeted cancer compounds. Consistent with our knowledge on the actionable pathways/gene alterations in HNSCC, targeted cancer compounds inhibiting the PI3K/AKT/MTOR (n = 44), EGFR (n = 28) and HDAC (n = 20) were found among the MOAs with the largest number of active compounds making up 10.5%, 7.5% and 4.8% of all active targeted compounds, respectively [Fig. 1C]. Of note, only 56% of all PI3K/AKT/MTOR inhibitors are active, indicating the variability in the efficacy of these inhibitors, while 82% of the EGFR inhibitors tested in PRISM primary screen are active in HNSCC. By contrast, all of the compounds targeting aurora kinase, PLK, protein synthesis and the proteasome are active in HNSCC. However, compounds of these MOAs are known to be extremely toxic in HNSCC since their targets are usually common essential genes. This can be seen where active compounds of these MOAs show killing effect across all or > 90% HNSCC lines (dark blue bar in Fig. 1C, with limited selectivity. Of the 3,062 non-oncology compounds, 404 (12%) were found to be active in HNSCC lines. The top MOAs included glucocorticoid receptor agonist (n = 13), cyclooxygenase inhibitor (n = 11) and adrenergic receptor agonist (n = 10) [Fig. 1D]. In the group of glucocorticoid receptor agonist, steroids such as hydrocortisone, prednisolone and methylprednisolone are among those active compounds. These non-oncology compounds had been widely used as palliative/supportive treatment or anti-inflammation agent for cancer patients^16^, and the current data suggest that they may also pose an anti-cancer effect that warrants further investigations.

More than half (n = 64, 66%) of the screened chemotherapy drugs are active in HNSCC, with the topoisomerase inhibitors (n = 20), tubulin polymerization inhibitor (n = 13) being the top MOAs [Fig. 1E]. The proportion of drugs being active in > 90% of HNSCC lines are also highest for these two MOAs, since they target essential cellular processes and are broadly toxic across all lines. Topoisomerase inhibitors that belong to anthracyclines drug such as doxorubicin, epirubicin, daunorubicin, nemorubicin, idarubicin all showed active killing in all tested lines. By contrast, the platinum-based chemotherapy including cisplatin and carboplatin that are widely used for HNSCC appears not as potent *in vitro*.

## Reference database and visualization of drug response profile of HNSCC

**Figure 2:**
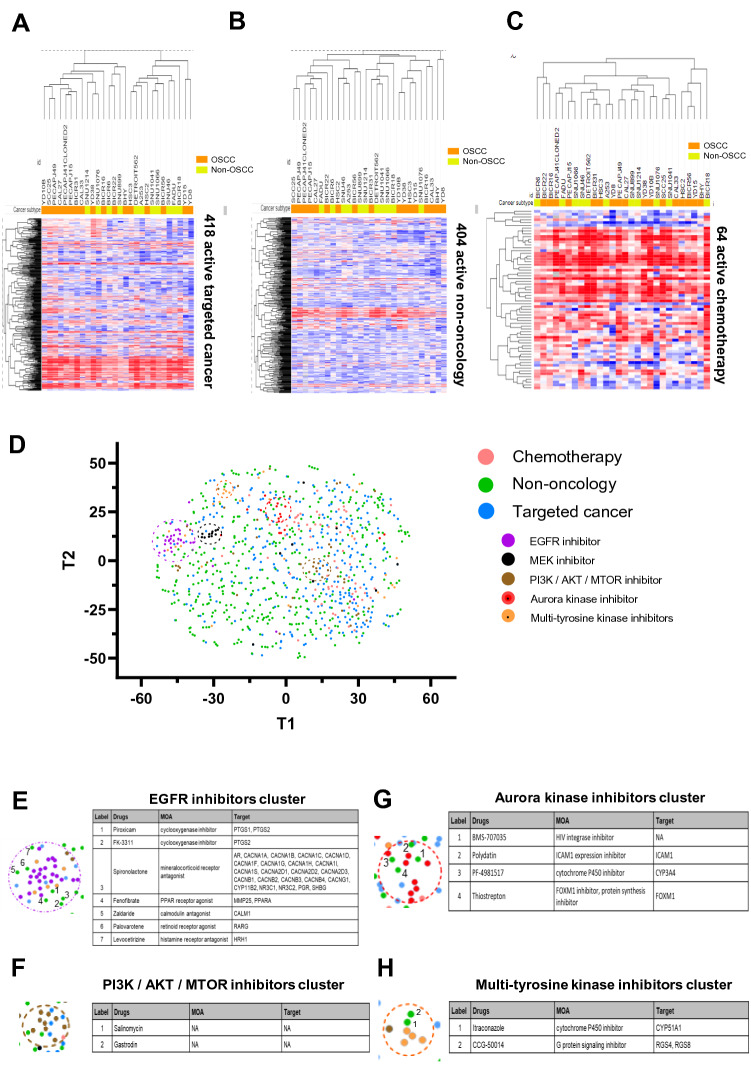
Visualization of drug response data using heatmap with hierarchical clustering and t-distributed stochastic neighbour embedding (tsne) analysis. Heatmap of drug response data from the PRISM primary screen on the 28 HNSCC cell lines, for (**A**) 418 active targeted cancer compounds; (**B**) 404 active non-oncology compounds; (**C**) 64 active chemotherapies. Heatmaps were plotted using Morpheus tool: https://software.broadinstitute.org/morpheus/. (**D**) High-dimensional reduction of drug response data of 886 active drugs in HNSCC was performed using tsne analysis (perplexity 30, iterations 100). Targeted cancer compounds that belong to the same top MOA classes such as PI3K/AKT/MTOR inhibitors, EGFR inhibitors, MEK inhibitors and Aurora kinase inhibitors cluster closely together. While generally the non-oncology compounds are very scattered across the plot, reflecting the likely diversity of MOAs. (**E**)–(**H**) Non-oncology compounds that consistently fall into the (**E**) EGFR inhibitor cluster, (**F**) PI3K/AKT/MTOR inhibitor cluster, (**G**) Aurora kinase inhibitor cluster and (**H**) Multi-tyrosine kinase inhibitor clusters.

To investigate the relationship between the drug response profile and cancer subtype (OSCC or non-OSCC) of HNSCC cell lines, we separated the PRISM screening compounds into three categories-targeted cancer compounds [Fig. 2A], non-oncology compounds [Fig. 2B] and chemotherapy [Fig. 2C]. From the unsupervised hierarchical clustering, cancer subtype (OSCC or non-OSCC) did not appear to be a distinguishing factor associated with overall drug response profiles. Moreover, there was no clear cluster of cell lines that share distinctive drug response patterns, when the cell lines are differentiated by their genomic alterations in key drivers of HNSCC [Fig. S2]. The high genetic heterogeneity of HNSCC cell lines, variability of response towards different drugs and the small number of cell lines could be possible reasons for this observation [Fig. S2]. For targeted compounds, the variability in response is seen across the majority of the drugs, suggestive of response based on genotype/targets status of each cell line. As expected, the non-oncology compounds are generally less potent across all HNSCC, but instead, their activities are rather selective in a small subset of cell lines [Fig. 2B]. On the contrary, most of the chemotherapy compounds are rather unselective and show potency across many of the HNSCC cell lines. Notably, the BICR18 line with a mismatch repair defect (*MSH2* and *MLH1*-mutated), appears to be sensitive to most of the chemotherapeutic agents [Fig. 2C].

To enable easier exploration and analysis to the interactive, customizable and searchable data matrix via the online Morpheus tool, we provide the data matrix for the 4,518 compounds × 28 HNSCC cell lines [Table S1] and the json files [Files S1–S3] for users to explore this data via Morpheus.

### Exploring the opportunity for repurposing of non-oncology drugs

Next, we investigated 404 active non-oncology compounds in HNSCC for drug repurposing for this disease. From the heatmap with hierarchical clustering, we noticed that active targeted compounds with clear and similar mechanisms of action often tend to cluster together, suggestive of consistent effect on cell lines and their putative activities were recapitulated in the screen. It has been previously shown that compounds with similar target profile and structure (and presumably similar MOA) clustered closely together when their drug sensitivity profiles are dimensionally reduced using tsne analysis^17^. As such, we performed tsne analysis [Fig. 2D] for unsupervised clustering of the 886 active compounds to identify any non-oncology compounds that show a similar killing pattern with those targeted compounds of distinct MOAs. Consistent with the Uniform manifold approximation and projection (UMAP) done by Corsello et al.^7^ on the pan-cancer cell lines, some of the targeted compounds consistently show strong clustering-this includes the EGFR inhibitors, MEKi, PI3K/AKT/MTOR inhibitors, multi-tyrosine kinase inhibitors (mTKIs) and Aurora kinase inhibitors [Fig. 2D]. Active compounds that kill > 90% of the HNSCC cell lines tend to cluster together, albeit not tightly and contains the majority of the less selective chemotherapy drugs.

Non-oncology compounds were very scattered across the plot, reflecting the vast diversity of MOAs and likely polypharmacology activities. Seven non-oncology compounds were clustered with the EGFR inhibitors (piroxicam, FK-3311, spironolactone, fenofibrate, palovarotene, levocetirizine) [Fig. 2E]. Among these, spironolactone and fenofibrate were also found in the cluster of EGFR inhibitors in Corsello’s pan-cancer UMAP^7^. Salinomycin and gastrodin were found to cluster closely with PI3K/AKT/MTOR inhibitors [Fig. 2F], whereas BMS-707035, polydatin, PF-4981517 and thiostrepton clustered with aurora kinase inhibitors [Fig. 2G]. Itraconazole and CCG-50014 are found within the mTKI cluster [Fig. 2H]. To facilitate researchers to navigate and generate new drug repurposing hypothesis in HNSCC, we have developed an interactive tsne plot akin to Fig. 2D and is available at https://anniechai.shinyapps.io/tsne/, [Fig. S3B]. Together with the interactive drug response data in the form of a heatmap matrix, we hope to provide a convenient and easily accessible reference database of active compounds for HNSCC.

### EGFR inhibitors are selectively enriched for killing activity in HNSCC

**Figure 3:**
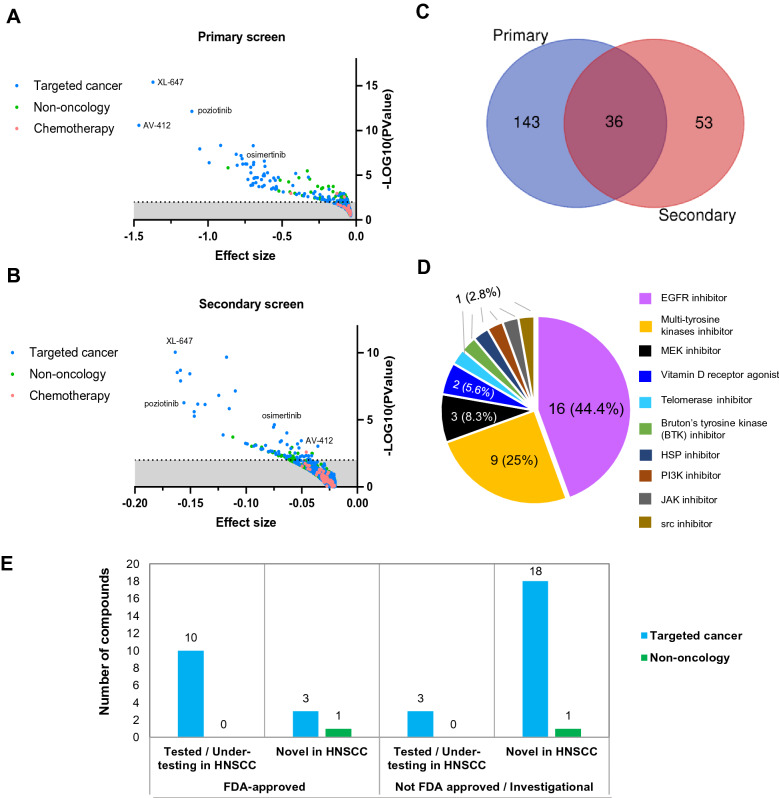
Identification of compounds that are selectively enriched in killing activity in HNSCC. (**A**) Using the “two-class comparison” function of the DepMap, differential analysis was conducted to compare drug response between HNSCC and non-HNSCC cell lines. Drug response data from the PRISM primary screen was used. Compounds that showed lower mean logFC in HNSCC (negative effect size) are plotted. *P* value < 0.01 or -log10(pvalue) > 2 is considered statistically significant. The top three most significant compounds that show preferential killing activity among HNSCC are XL-647, AV-412 and poziotinib. (**B**) Compounds with preferential sensitivity in HNSCC, based on PRISM secondary screen data. (**C**) Venn diagram of significantly selective compounds identified from primary and secondary screen shortlisted 36 common compounds (**D**) Of the 36 shortlisted compounds, mainly are EGFR inhibitors (n = 16, 44.4%), multi-tyrosine kinase inhibitors (n = 9, 25%) and MEK inhibitors (n = 3, 8.3%). (**E**) Classification of the 36 compounds based on their drug category, FDA approval status and novelty. Among these, ten targeted cancer compounds had been tested in HNSCC, including several EGFR inhibitors such as afatinib and gefitinib, as well as MEK inhibitors such as trametinib and cobimetinib. On the other hand, three targeted cancer compounds (osimertinib, neratinib and bosutinib) are potentially good novel candidates for drug repurposing as these are FDA-approved, but have yet to be tested on HNSCC in clinical trials. The majority of the compounds fall into the category of non-FDA approved and have never been tested in HNSCC (n = 18 targeted therapy and n = 1 non-oncology).

The full PRISM primary dataset included 578 cell lines (24 tumour types)^7^, by comparing HNSCC to all other non-HNSCC cell lines, we found 179 compounds that are significantly more effective in HNSCC (− log10(*P*-value) > 2 or *P*-value < 0.01). About half of the significantly enriched drugs are targeted therapy (n = 90, 49.7%), with the top three most significant drugs being XL-647, poziotinib and AV-412 [Fig. 3A]. Non-oncology drugs made up nearly the other half (n = 88, 48.6%), and only three were chemotherapy compounds (n = 3, 1.7%) [Table S2]. To strengthen our analysis, we also analyzed the PRISM secondary screen data. Consistently, the top three most significant compounds are also found in the secondary screen, together with 86 other compounds [Fig. 3B] [Table S3]. In total, 36 compounds are consistently found to be selectively effective in HNSCC in both the primary and secondary screens [Fig. 3C]. Analysis of the MOAs of these compounds revealed that nearly half (n = 16, 44.4%) are EGFR inhibitors, followed by nine mTKIs and three MEKi [Fig. 3D]. The observation that EGFR inhibitors are significantly enriched and more effective in HNSCC compared to other non-HNSCC is consistent with the observation that EGFR gene dependency is also selectively enriched among HNSCC [Fig. S4A]. A previous HNSCC-specific analysis of the GDSC also found EGFR inhibitors (afatinib and gefitinib) among the four drugs (50%) with significantly higher sensitivity in HNSCC^18^.

### FDA approved drugs with promising killing activity in HNSCC

**Figure 4:**
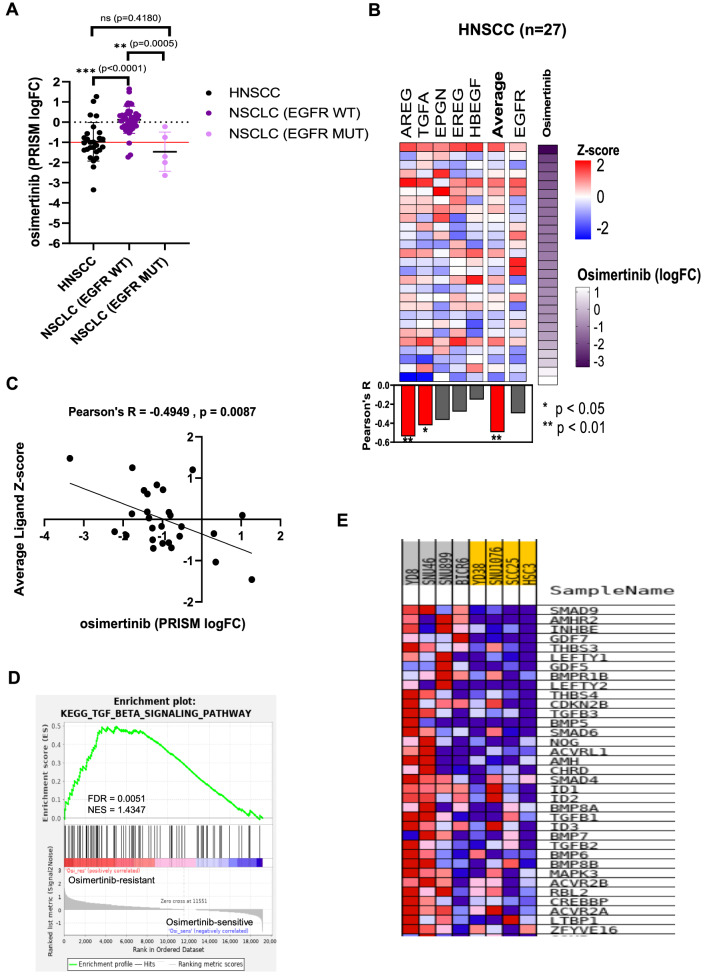
Identification of biomarker of response for osimertinib. (**A**) The mean logFC from the PRISM primary screen for osimertinib in HNSCC is comparable with NSCLC with EGFR mutations (*P* = 0.4180). Both the clinically responsive subset (NSCLC with EGFR mutation) (*P* = 0.0005) and unselected HNSCC (*P* < 0.0001) have mean logFC that are significantly lower than the subset of NSCLC without EGFR mutation. (**B**) Pearson’s correlation of EGFR ligands (AREG, TGFA, EPGN, EREG and HBEGF) or EGFR gene expression with osimertinib sensitivity in 28 HNSCC cell lines (each row is a cell line). (**C**) Average EGFR ligands expression (Z-score) is significantly correlated with osimertinib sensitivity (logFC) (Pearson’s R = -0.4949, *P* = 0.0087). (**D**) Gene set enrichment analysis (GSEA) reveal significant upregulation of the TGF-beta signalling pathway among the osimertinib-resistant cell lines. (**E**) Gene expression heatmap from GSEA, showing the up-regulated genes within the TGF-beta signalling pathway. (**A**) to (**C**) were plotted using GraphPad Prism software 8.0.2. (**D**) and (**E**) were figures generated from running the GSEA modules from GenePattern 2^11^ (https://www.genepattern.org/).

Among the 36 compounds that are selectively enriched in HNSCC, 14 (39%) are already FDA-approved for other cancers [Fig. 3E] [Table S4]. Of the 22 (61%) non-FDA approved drugs, 19 were considered “novel” as they have not been tested in HNSCC before [Fig. 3E] [Table S4]. There are three compounds (osimertinib, neratinib, and bosutinib) that represent promising novel candidates for drug repurposing, as these are FDA-approved, but not tested in clinical trials on HNSCC patients. Osimertinib is approved for EGFR-mutated NSCLC (e.g. exon 19 deletions, p.L858R and p.T790M). However, these EGFR mutations are rare in HNSCC^19^. Interestingly, when we compare the drug response data of unselected HNSCC versus that of NSCLC with or without the actionable mutations, HNSCC sensitivity towards osimertinib was comparable with that of EGFR-mutated NSCLC (*P* = 0.4180), and also significantly more sensitive than EGFR-wildtype NSCLC (*P* < 0.0001) [Fig. 4A]. Some of the HNSCC cell lines are even more responsive than the NSCLC with EGFR mutations. This suggests that despite the lack of actionable mutation in EGFR, HNSCC are still very dependent on EGFR signalling. In fact, osimertinib treatment resulted in a significant antitumor effect in an *in vivo* study using two EGFR-wildtype HNSCC models (FADU and CAL27)^45^. Therefore, the repurposing of osimertinib to inhibit this essential EGFR signalling in HNSCC is promising. Unlike in NSCLC, where activating mutation, high EGFR expression (Pearson’s R = − 0.3675, *P* = 0.0120) and dependency (Pearson’s R = 0.4757, *P* = 0.105) were associated with osimertinib sensitivity, EGFR expression (Pearson’s R = 0.2957, *P* = − 0.1341) and dependency (Pearsons’ R = 0.2723, *P* = 0.1980) were not associated with osimertinib sensitivity in HNSCC [Fig. S4B–C]. However, further mechanistic investigation is needed to delineate the mode of action and to identify any relevant biomarker in the HNSCC context.

### Identification of HNSCC tissue-specific biomarker of response for osimertinib

A recent proteogenomics study of HNSCC has proposed two models of EGFR activation: ligand-dependent and ligand-independent pathways^20^. In the context of HNSCC, the ligand-dependent mode is often observed, where the availability of EGFR ligands is the rate-limiting factor where the abundance of EGFR ligands (*AREG, EREG, HBEGF, EPGN* and *TGFA*), are associated with EGFR pathway activation and response towards cetuximab in the patient-derived xenograft (PDX) models^20^. Hence, we examined if the sensitivity towards osimertinib can also be better predicted by the EGFR ligands abundance. We correlated the expression levels of the five EGFR ligands individually, as well as their average expression, with osimertinib sensitivity [Fig. 4B]. We found that the average expression of all EGFR ligands, instead of EGFR expression, is significantly correlated with the osimertinib sensitivity [Fig. 4C], corroborating the recent proteogeonomic findings. In contrast, in NSCLC (ligand-independent mode), the *EGFR* expression is significantly correlated with osimertinib sensitivity [Fig. S4D]. This further highlight the tissue-specific difference in the mechanism underlying drug sensitivity and biomarker selection.

Development of resistance towards tyrosine kinase inhibitor is common, but an earlier understanding of intrinsic resistance could shed light on how to manage unresponsiveness or acquired resistance. We performed GSEA on the four most sensitive and four most resistant lines to identify activated pathways that might govern the response towards osimertinib [Table S5]. The TGF-beta signaling pathway is significantly enriched (normalized enrichment score = 1.4347; nominal p-value = 0.0051) among the resistant lines [Fig. 4D]. Significantly up-regulated genes included the ligands of TGF-beta receptors (*TGFB1, TGFB2,* and *TGFB3*), as well as the effector proteins (*SMAD4, SMAD6,* and *SMAD9*) and some of its target genes (*ID1, ID2* and *ID3*) [Fig. 4E].

### Uncovering biomarker of response/resistance for MEKi

We found three MEKi (trametinib, cobimetinib and AZD8330) among the compounds that were enriched for killing activity in HNSCC when compared to non-HNSCC [Fig. 2D]. Trametinib and cobimetinib (in combination with vemurafenib) have been approved to treat unresectable or metastatic melanoma with BRAF V600E or V600K mutations^21^. Although BRAF mutations are rare in HNSCC, mutations in the MAPK pathway are found in 18.6% of HNSCC^22^, suggesting that targeting MAPK pathway could be a promising strategy in HNSCC. Trametinib has only been tested in a window-of-opportunity trial [NCT01553851], where it resulted in a median of 46% reduction in tumour size among 17 HNSCC patients^23^. However, the biomarkers that were being investigated (p-ERK1/2 and CD44) did not correlate well with response^23^.

**Figure 5:**
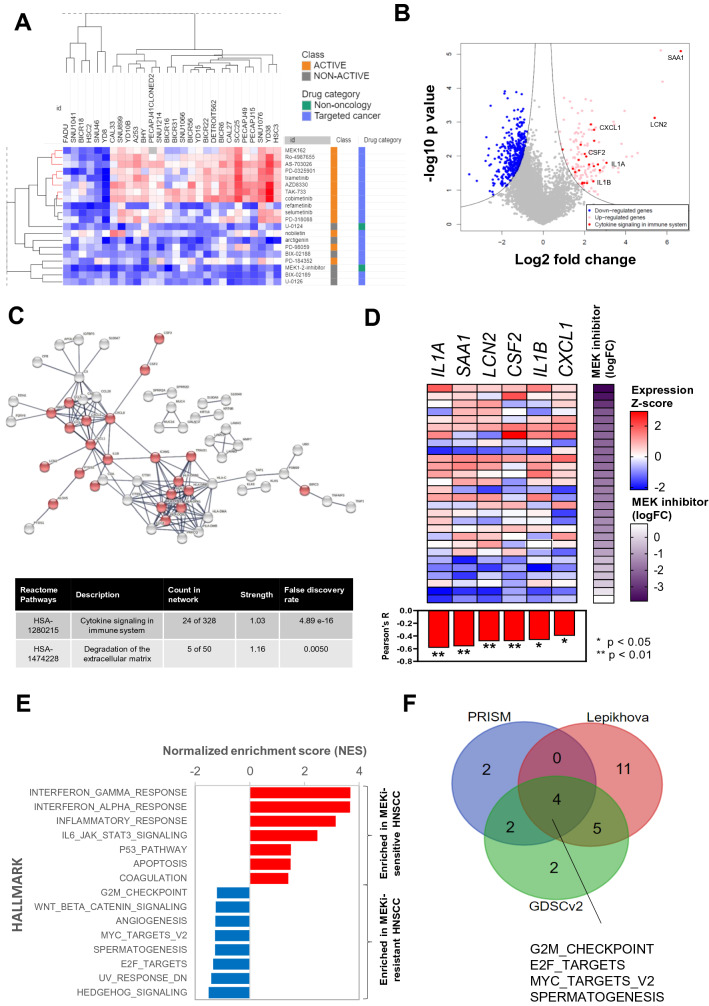
Uncovering candidate biomarkers of response and possible mechanism of intrinsic resistance towards MEKi. (**A**) Heatmap (generated using Morpheus tool: https://software.broadinstitute.org/morpheus/) with hierarchical clustering showing the drug sensitivity profile of 28 HNSCC cell lines towards all 20 MEKi. Some subclusters (in red) consisting of eight MEK inhibitors showed a largely similar pattern of sensitivity. (**B**) Volcano plot of differentially expressed genes between MEKi-sensitive and MEKi-resistant HNSCC. (**C**) STRING network analysis of 136 significantly upregulated genes among the MEKi-sensitive cell lines, revealed enrichment of REACTOME pathway (HSA-1280215-“Cytokines signalling in immune system” [highlighted in red]. A total of 24 genes were in this Reactome pathway (unconnected nodes are hidden). (**D**) Pearson’s correlation between the gene expression of six cytokines (IL1A, SAA1, LCN2, CSF2, IL1B and CXCL1) with significant correlation with the average potential drug sensitivity against MEKi (n = 28). Graph was plotted using GraphPad Prism software 8.0.2. (**E**) GSEA analysis of MEKi-sensitive and MEKi-resistant cell lines, with immune-related hallmark pathways being enriched among MEKi-sensitive HNSCC; While in MEKi-resistant HNSCC, hallmarks that are enriched are proliferation or cell cycle-related pathways. (**F**) Comparison of enriched hallmarks among the MEKi-resistant HNSCC, from GDSCv2 and Lepikhova datasets. The hallmarks of E2F_Targets, MYC_Targets_V2, G2M_checkpoint and spermatogenesis are consistently associated with MEKi resistance.

We next sought to explore potential biomarkers of MEKi in HNSCC. Of the 20 MEKi in PRISM, 14 (70%) were active compounds in HNSCC. Unsupervised hierarchical clustering showed a distinct cluster consisting of eight active MEKi (red branches) [Fig. 5A] [Table S6]. Six cell lines were distinctively non-responsive towards all the MEKi, while the other 22 cell lines show variable response. To identify potential gene expression signature or single gene marker that may predict response, we performed DEG analysis between the six most sensitive and six most resistant cell lines. We identified 136 significantly up-regulated genes among the MEKi-sensitive HNSCC, and 428 significantly down-regulated genes [Fig. 5B] [Table S7]. Among the 136 genes, significant enrichment of genes in the Reactome pathway of “Cytokine Signalling in Immune System” (HSA-1280215) was observed. As shown in the STRING functional protein association analysis [Fig. 5C], 24 of the genes in this pathway are significantly up-regulated and interconnected. Among these, we further shortlisted six cytokines (*IL1A, SAA1, LCN2, CSF2, IL1B* and *CXCL1*), where their gene expression significantly correlated with MEKi sensitivity across the 28 HNSCC cell lines [Fig. 5D]. To complement the DEG analysis, we also performed GSEA and showed that immune-related pathways such as the interferon gamma/alpha responses and inflammatory response hallmark pathways were significantly enriched among the MEKi-sensitive group [Fig. 5E]. On the other hand, proliferation or cell-cycle related hallmark pathways such as the G2M checkpoint, MYC targets (v2) and E2F targets are significantly enriched among the MEKi-resistant group [Fig. 5E]. To confirm our findings, we performed GSEA on MEKi-resistant HNSCC from the GDSC (v2) and Lepikhova et al. dataset [Table S8]. G2M checkpoint, MYC targets (v2), E2F targets and spermatogenesis were commonly enriched hallmark pathways [Fig. 5F]. This further substantiates that aberrantly activated cell cycle and pro-proliferative pathways are associated with resistance towards MEK inhibition. Up-regulation of genes such as CCND1, a critical cell-cycle regulator is observed among the MEKi-resistant HNSCC. CCND1 overexpression is known to promote tumorigenesis in various cancers, and CCND1 amplification is a common event in HNSCC resulting in the dysregulation of cell cycle pathways. The CCND1-CDK4/6 complex phosphorylates Rb and releases E2F into the nucleus, leading to transcriptional activation of downstream E2F targets^24^. Whilst clinical trials utilizing CDK4/6 inhibitors are underway, this data suggests that a combination of CDK4/6 inhibitors such as palbociclib and abemaciclib with MEKi could be an effective strategy to overcome intrinsic resistance towards MEKi in HNSCC.

## Discussion

The increased availability of high-throughput pharmacogenomics data have provide an opportunity to perform drug repurposing in specific cancer type. Here, we have performed a systematic in silico analysis on the PRISM drug screening data to identify compounds for HNSCC. We demonstrated that some of the non-oncology compounds could be exploited for this devasting disease. More importantly, we identified and validated the findings using independent data sets to gain insights the MOA of these compounds and their potential biomarkers for HNSCC.

Our HNSCC-specific analysis of the PRISM dataset has uncovered a rich resource of targeted compounds that holds the potential to be repurposed, and also revealed a large number of active non-oncology compounds that could have activity in HNSCC. Despite new anti-cancer drugs being tested on HNSCC patients, most fail to show clear clinical benefit. This could be due in part to the absence of biomarkers for patients stratification, as the use of biomarkers was associated with a four-fold increase in the success rates for oncology clinical trials^25^. One of the unique propositions of this dataset is that all 28 HNSCC lines have deep molecular characterization, where genomics, transcriptomics, epigenomics, proteomics and essentialomics (essential genes profile from CRISPR/Cas9 screens) data are available^7^. Integration of multi-omics data could enable the identification of biomarker of response and improve our understanding of drug MOAs and potential resistance mechanism. Among these features, the transcriptomics (mRNA expression) was found to be most predictive of drug sensitivities^7,26^. Our findings shed light on the development of gene-expression based biomarker for HNSCC, especially where mutations in actionable genes are relatively rare in this cancer type.

A limitation while interpreting this dataset is that this PRISM primary screen is done at a single dose of 2.5 µM. Some highly potent compounds might have saturated anti-cancer effect at this dose and cannot be differentiated from those that are less potent. Further, the use of logFC relative to the DMSO control as a surrogate marker of drug sensitivity, instead of IC50 or EC50 (half-maximal effective concentration), limits our ability to determine the potency of each of the compounds. While this could be overcome by examining the secondary PRISM screen, there was a substantial reduction in the number of compounds tested (4,518 in primary screen vs 1,207 in secondary screen) and only 22 HNSCC lines were included. Furthermore, there were missing data points in the secondary screen where, of the expected 1,207 × 22 data matrix, only 60% of the data points were available [Fig. S5]. Hence, the secondary screen data was used only for validating the data from the primary screen. Furthermore, due to the pooling of a mixture of molecular-barcoded cancer cell lines, the drug response might be confounded by paracrine-mediated modulation^6,7^.

Whilst an inherent limitation of high-throughput drug screening precludes the role of the stromal and immune compartments, known clinical responses were well recapitulated in this PRISM dataset, demonstrating the utility and relevance of cancer cell line-derived drug response data. Large-scale analysis found that oncogenic alterations in tumour tissue can be faithfully recapitulated by cancer cell lines^5^. Besides, a recent pan-cancer study called the Celligner, revealed that HNSCC cell lines (which included the 28 lines tested in PRISM) rank second after Ewing sarcoma, where the transcriptomics profile of cell lines and patient tumours are highly correlated^27^. In the HNSCC dataset, we identified possible FDA-approved drug candidates including osimertinib and trametinib that could be evaluated for the treatment of HNSCC. Further, we also offer insights into the potential candidates of non-oncology compounds to be repurposed for HNSCC, which can be explored further with our RShiny app or the interactive heatmap.

Using tsne projection of the drug sensitivity profile [Fig. 2D], some non-oncology drugs showed similar activities with subsets of targeted compounds with defined MOAs. Piroxicam and FK-3111 which are non-steroidal anti-inflammatory drugs (NSAIDs) approved for treating pain and inflammation, are two examples of cyclooxygenase inhibitors that clustered closely with EGFR inhibitors. Epidemiological studies have reported associations of NSAIDs use with reduced cancers risk, thus prompting the investigation of their anti-cancer properties^28^. Piroxicam is used widely to treat OSCC in dogs^29^, its anti-cancer properties have also been demonstrated in several human cancers^30–32^. In pre-malignant and malignant human oral cancer lines, piroxicam selectively inhibited malignant cell growth via cell cycle arrest in the S phase^33^.

Another interesting non-oncology drug is salinomycin, that clustered with PI3K/AKT/MTOR inhibitors. Salinomycin is widely used as an anticoccidial drug in poultry but was later found to be a potent and selective inhibitor of epithelial cancer stem cell (CSC) growth from a high-throughput screen^34^. Salinomycin preferentially inhibits breast CSCs at a potency of more than 100-fold that of paclitaxel and resulted in potent suppression of tumour growth *in vivo*^34^. Subsequently, numerous studies showed that salinomycin is effective as an anti-cancer drug in various cancers^35–38^. In HNSCC, salinomycin treatment resulted in a 71.5% increase in apoptosis and significantly reduced the sphere-forming ability of HNSCC CSCs^39^. Interestingly, salinomycin was shown to induce phosphorylation of AKT, this is consistent with the effect of specific AKT inhibitors when tested in HNSCC^40,41^. This further suggests that salinomycin may share similar MOA with the AKT inhibitors, as indicated by their clustering in the tsne. Notably, salinomycin (specifically HSB-1216, delivered via QUATRAMER technology) has been granted orphan drug designation by the FDA in 2020, and a Phase I trial is planned for small cell lung cancer^42–44^. Further investigation would be of interest to uncover the potential utility of salinomycin for HNSCC treatment, and our discovery here may shed light into uncovering its MOA and enabling selection of patients with biomarker of response. To facilitate more discovery research, our interactive heatmap and the tsne plot on RShiny app (https://anniechai.shinyapps.io/tsne/) would serve as a useful starting point.

Importantly, from the thousand of compounds screened, we have shortlisted 36 promising compounds [Fig. 3C–E] that should be prioritized for further investigation, due to their enriched killing activities among HNSCC lines. Although EGFR is an enriched dependency in HNSCC and EGFR inhibitors are most active in killing HNSCC relative to other cancers, Cetuximab is the only approved targeted therapy for use in HNSCC. In this study, we focused on osimertinib, a third-generation EGFR inhibitor approved for NSCLC patients with exon 19 deletions, L858R and T790M point mutations in EGFR. Despite not having these mutations, osimertinib was found to be equally effective in HNSCC, with no significant difference in logFC when compared to the EGFR mutation-positive NSCLC. In HNSCC, EGFR expression/amplification is not predictive of response to EGFR inhibitors^20,45,46^. Consistent with more recent studies on cetuximab and panitumumab, we have demonstrated that the expression of the EGFR ligands but not EGFR is more predictive of osimertinib response. This supports the notion that in HNSCC, the rate-limiting factor governing EGFR pathway activation is the expression of EGFR ligands (ligand-dependent pathway)^20^; while in another context such as NSCLC, the EGFR ligand-independent pathway might be prevailing^47^. Using GSEA, we found that activation of TGF-beta signalling might play a role in mediating resistance towards osimertinib. The use of TGF-beta blocking antibody diminishes the emergence of cetuximab-resistant HNSCC^48^ and TGF-beta activated CAFs were shown to suppress cetuximab activity in HNSCC *in vitro* and *in vivo* PDX models^49^ demonstrating that integrative analysis of drug response and genomics features in cancer models could identify mechanisms underlying response to cancer therapeutics. Although a preclinical study has shown the efficacy of osimertinib as a single agent in inhibiting *in vivo* tumour growth^50^, no clinical trial yet has tested osimertinib in HNSCC. Our data suggests that osimertinib could be efficacious in HNSCC expressing high levels of EGFR ligands, with low expression of members of the TGF-beta pathway, and clinical trial designs that examine and validate these biomarkers is warranted.

Another class of targeted cancer compounds with promising efficacy in HNSCC is MEKi. Clear selectivity of MEKi are seen where subsets of HNSCC cell lines demonstrated distinct responses to MEKi suggesting that predictive markers of response are crucial for patient selection. Unlike in melanoma, where BRAF mutation status could help to identify responders to vemurafenib, other mechanisms of response must be at play in HNSCC as BRAF mutations are uncommon in HNSCC^51^. Shared mutations among the six most resistant lines were not found, highlighting that the lack of response could be heterogeneous and likely not driven by mutations. Emerging studies have revealed that transcriptomic profiles could be more powerful in predicting drug responses compared to mutational profiles in some context^7,26^. From our DEG analysis, we revealed that up-regulation of several cytokines and immune-related pathways are associated with sensitivity towards MEKi. This is consistent with the increasingly recognized immunomodulatory roles of the MAPK pathway in cancers^52^. Among these are interleukin genes such as the *IL1A* and *IL1B*, which are pro-inflammatory cytokines that could induce activation of the MAPK pathway^53^ Besides, *SAA1, CXCL1* and *CSF2* have also been implicated to regulate the MAPK pathway^54^. HNSCC has been shown to be one of the most immune-enriched solid tumors with the majority belonging to the IFNγ-dominant immune subtype^55^. The upregulation of pro-inflammatory cytokines that confers sensitivity towards MEK inhibition could therefore explain why a majority of HNSCC cell lines are responding to MEKi despite the lack of activating mutations in the Ras/Raf/MAPK pathways. This concept is currently being tested in an on-going clinical trial [NCT03264066] of combining cobimetinib with atezolimumab (anti-PDL1 mAb) in HNSCC and other cancers. Some HNSCC however, were intrinsically resistant towards MEK inhibition. In this regard, we found cell cycle and proliferation-related pathways to be consistently associated with resistance towards MEKi. This supports the combination of CDK4/6 inhibitors with MEKi for better synergy, which has been investigated in other cancers, such as melanoma, colorectal and NSCLC^56^. However, these were investigated in the context of the presence of mutation(s) in the Ras/Raf/MAPK pathway which is lacking in HNSCC. Instead, in HNSCC, we showed that the immune-cytokines enriched HNSCC could promote responsiveness to MEKi. The potential utility of those shortlisted biomarkers of response, as well as the efficacy of combining MEKi with CDK4/6 warrant further investigation in HNSCC.

## Conclusions

In summary, our HNSCC-specific analysis of the PRISM repurposing dataset has helped to reveal the targetable MOAs within HNSCC and shortlisted 36 drug candidates to prioritize for repurposing in HNSCC. Our processed HNSCC drug response dataset that is made available in the form of interactive heatmaps or RShiny app webpage is an important resource that could accelerate drug development in the context of HNSCC. We have also identified several HNSCC-specific biomarkers for EGFR inhibitor and MEKi that warrant further validation. Furthermore, using transcriptomic-based pathway-level enrichment analysis, we demonstrated how rational drug combination may be proposed, such as in the case of MEKi and CDK4/6 inhibitor. These exemplify the potential of our study in enabling new discoveries in identifying promising new drugs and biomarkers for further investigation, to expand the treatment options for HNSCC. In addition, our in silico analysis pipeline can also be applied to other cancers covered in this PRISM dataset, for their corresponding drug repurposing exploration.

## Supplementary Information

Supplementary Information 1.

Supplementary Information 2.

Supplementary Information 3.

## Data Availability

The full PRISM primary screen dataset used in this study is available from the Broad Institute Cancer Dependency Map (DepMap) portal [RRID: SCR_017655] (https://depmap.org/repurposing/-“primary_replicate_collapsed_logfold_change.csv”, 19Q4). The identities of the cell lines were mapped using the “primary-screen-cell-line-info.csv” file, while the information of the compounds tested were mapped using the “2- Compounds tested” tab from “Corsello_supplemental_tables.xlsx”. Supplementary Files S1–S4 are downloadable from figshare via-https://doi.org/10.6084/m9.figshare.16443759.v1.
