# Supplementary material for: Uncovering drug repurposing candidates for head and neck cancers: insights from systematic pharmacogenomics data analysis
Source: Sci Rep. 2021 Dec 14;11:23933. doi: 10.1038/s41598-021-03418-1 (PMC8671460; doi:10.1038/s41598-021-03418-1)
Supplement: Supplementary file 1 — Supplementary Information 1. [file 41598_2021_3418_MOESM1_ESM.pptx]

## Slide 1
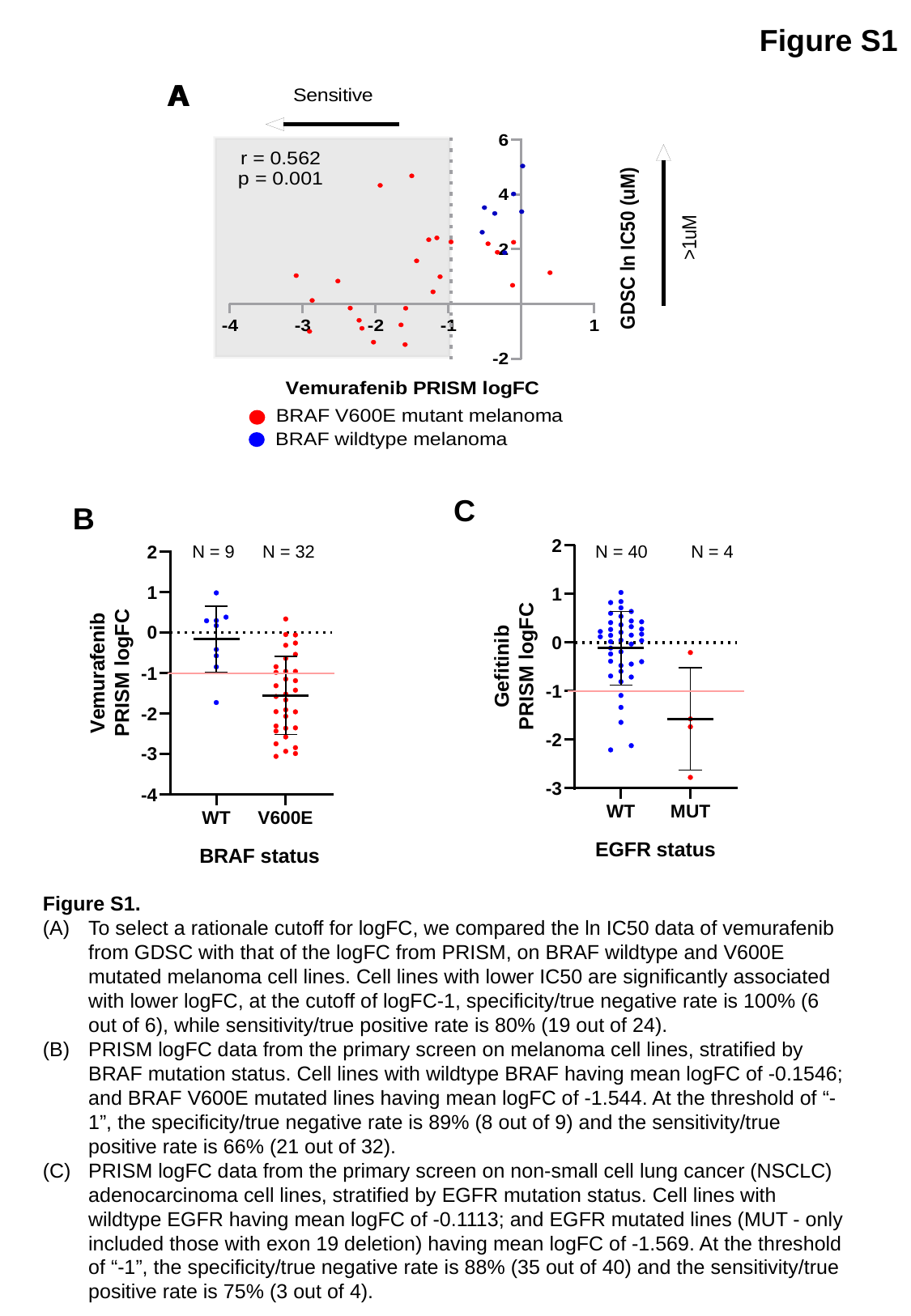

Figure S1
A
A
C
B
N = 9
N = 32
N = 40
N = 4
Figure S1.
To select a rationale cutoff for logFC, we compared the ln IC50 data of vemurafenib from GDSC with that of the logFC from PRISM, on BRAF wildtype and V600E mutated melanoma cell lines. Cell lines with lower IC50 are significantly associated with lower logFC, at the cutoff of logFC-1, specificity/true negative rate is 100% (6 out of 6), while sensitivity/true positive rate is 80% (19 out of 24).
PRISM logFC data from the primary screen on melanoma cell lines, stratified by BRAF mutation status. Cell lines with wildtype BRAF having mean logFC of -0.1546; and BRAF V600E mutated lines having mean logFC of -1.544. At the threshold of “-1”, the specificity/true negative rate is 89% (8 out of 9) and the sensitivity/true positive rate is 66% (21 out of 32).
PRISM logFC data from the primary screen on non-small cell lung cancer (NSCLC) adenocarcinoma cell lines, stratified by EGFR mutation status. Cell lines with wildtype EGFR having mean logFC of -0.1113; and EGFR mutated lines (MUT - only included those with exon 19 deletion) having mean logFC of -1.569. At the threshold of “-1”, the specificity/true negative rate is 88% (35 out of 40) and the sensitivity/true positive rate is 75% (3 out of 4).

## Slide 2
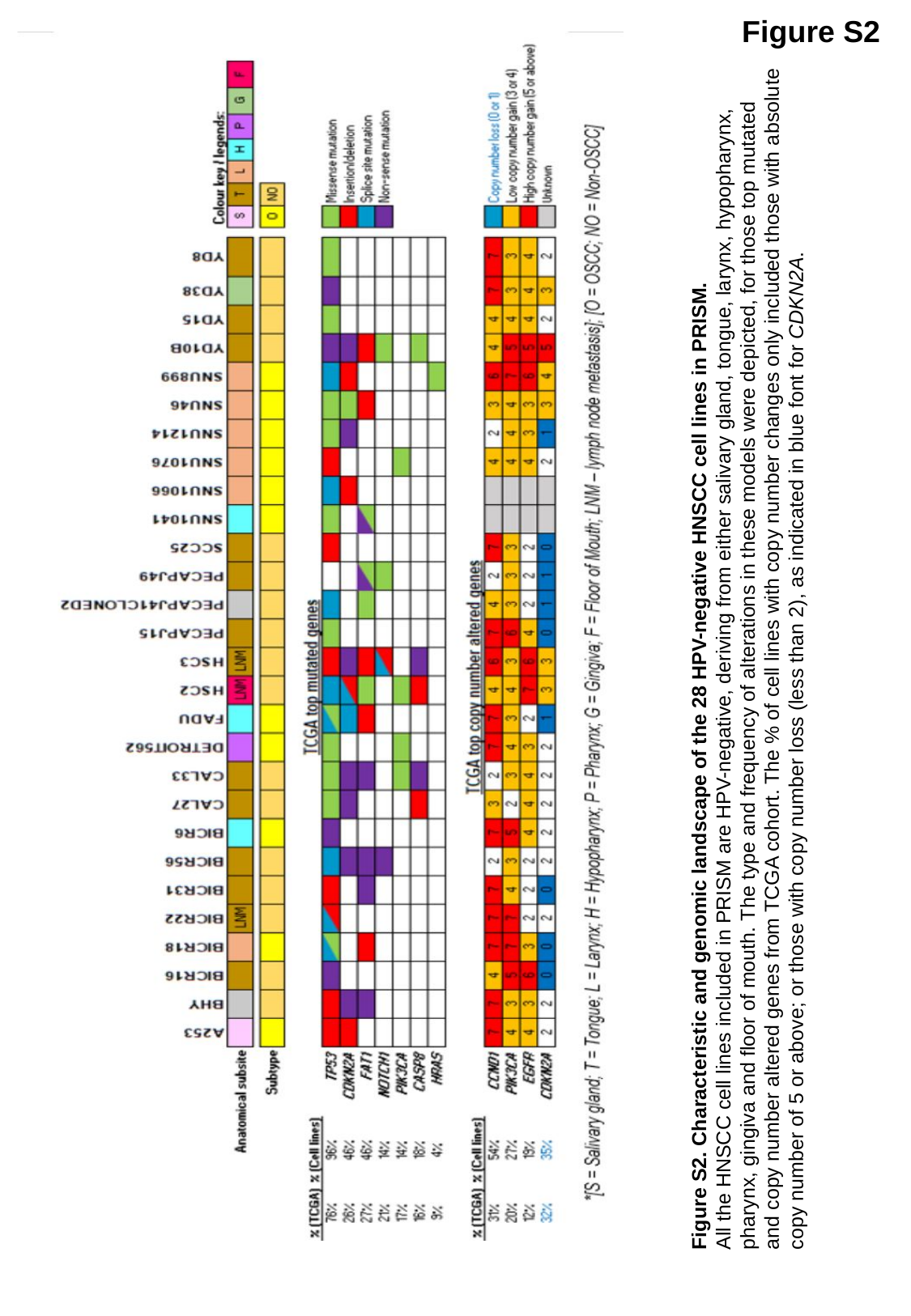

Figure S2
Figure S2. Characteristic and genomic landscape of the 28 HPV-negative HNSCC cell lines in PRISM.
All the HNSCC cell lines included in PRISM are HPV-negative, deriving from either salivary gland, tongue, larynx, hypopharynx, pharynx, gingiva and floor of mouth. The type and frequency of alterations in these models were depicted, for those top mutated and copy number altered genes from TCGA cohort. The % of cell lines with copy number changes only included those with absolute copy number of 5 or above; or those with copy number loss (less than 2), as indicated in blue font for CDKN2A.

## Slide 3
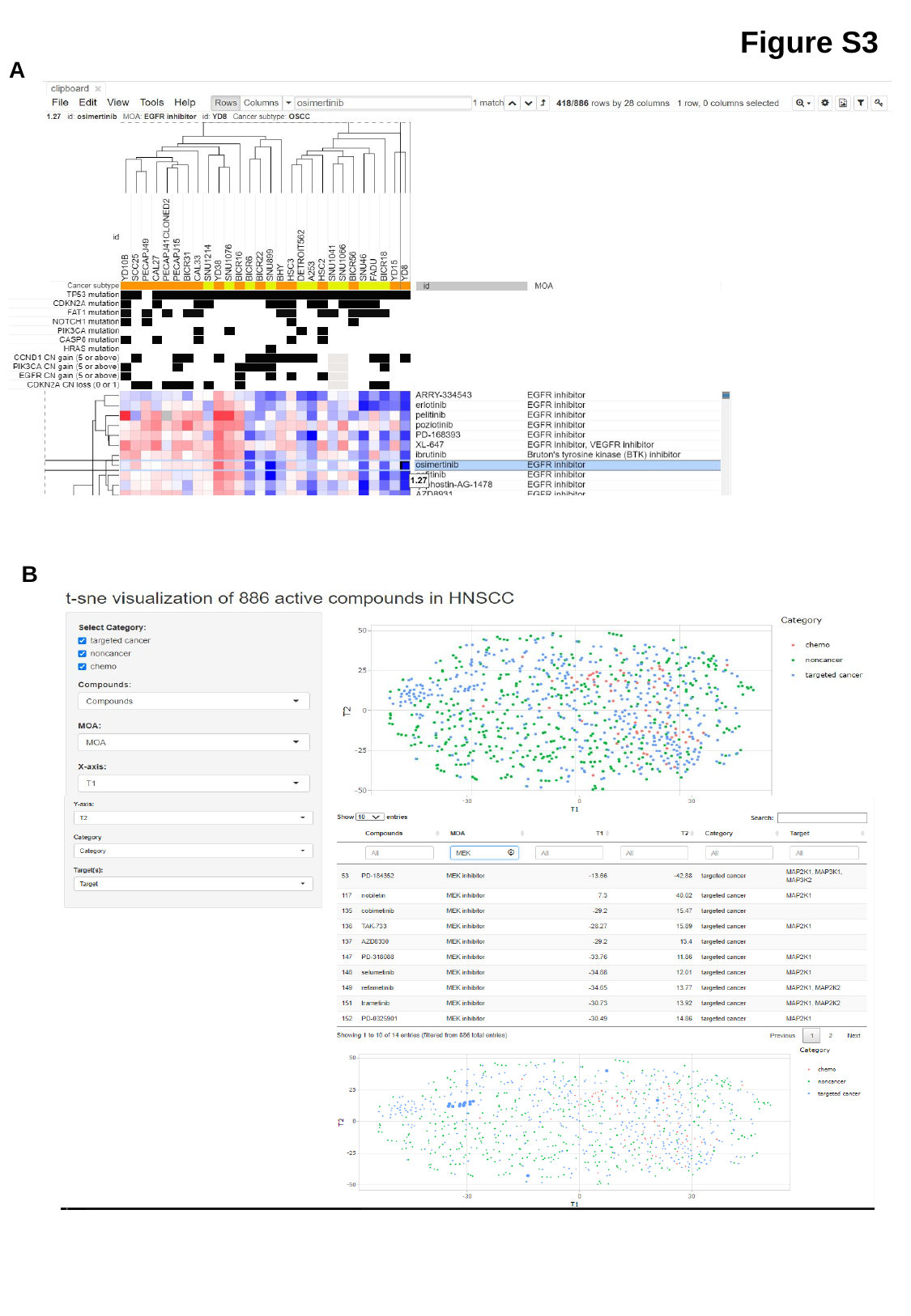

Figure S3
A
B

## Slide 4
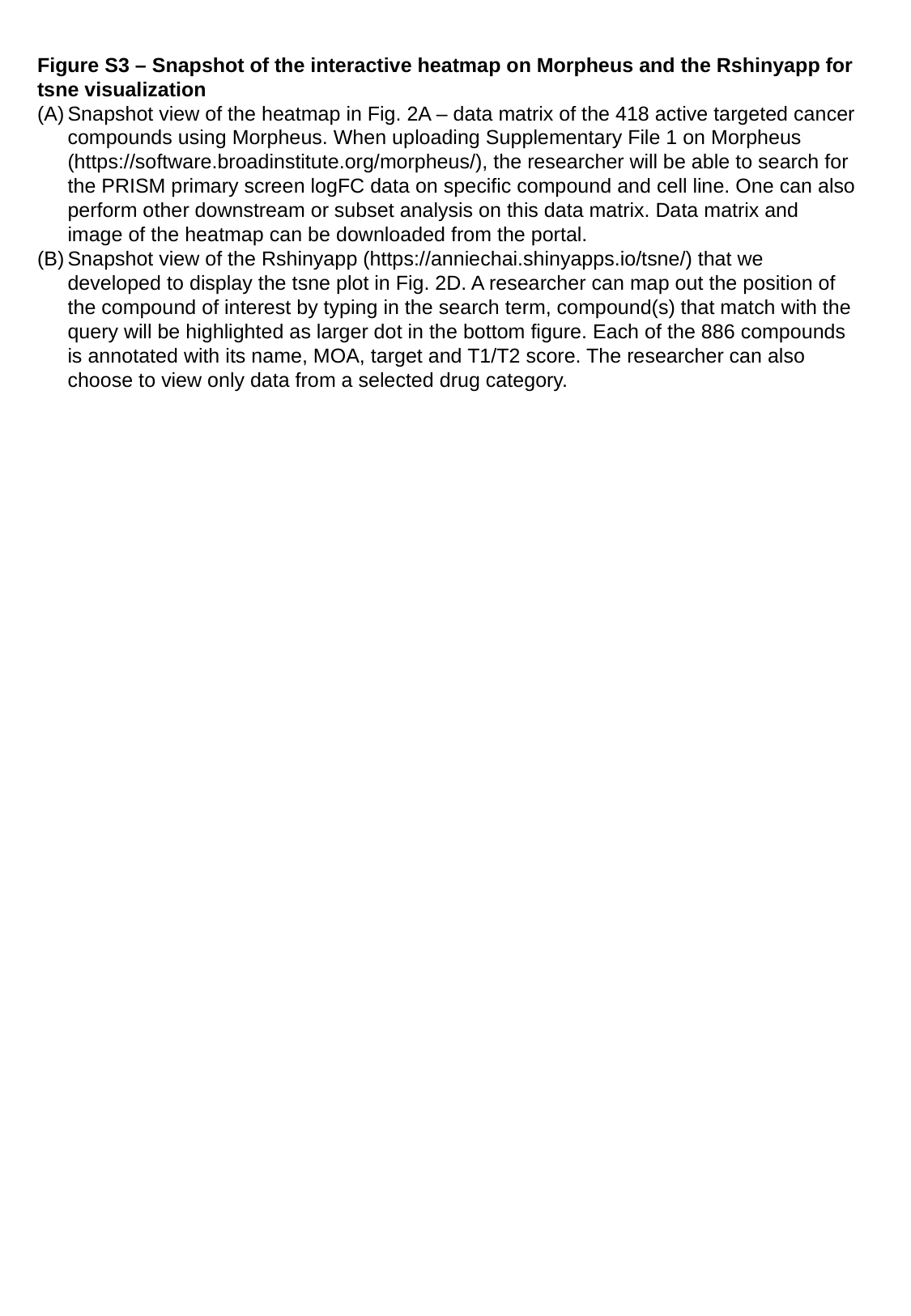

Figure S3 – Snapshot of the interactive heatmap on Morpheus and the Rshinyapp for tsne visualization
Snapshot view of the heatmap in Fig. 2A – data matrix of the 418 active targeted cancer compounds using Morpheus. When uploading Supplementary File 1 on Morpheus (https://software.broadinstitute.org/morpheus/), the researcher will be able to search for the PRISM primary screen logFC data on specific compound and cell line. One can also perform other downstream or subset analysis on this data matrix. Data matrix and image of the heatmap can be downloaded from the portal.
Snapshot view of the Rshinyapp (https://anniechai.shinyapps.io/tsne/) that we developed to display the tsne plot in Fig. 2D. A researcher can map out the position of the compound of interest by typing in the search term, compound(s) that match with the query will be highlighted as larger dot in the bottom figure. Each of the 886 compounds is annotated with its name, MOA, target and T1/T2 score. The researcher can also choose to view only data from a selected drug category.

## Slide 5
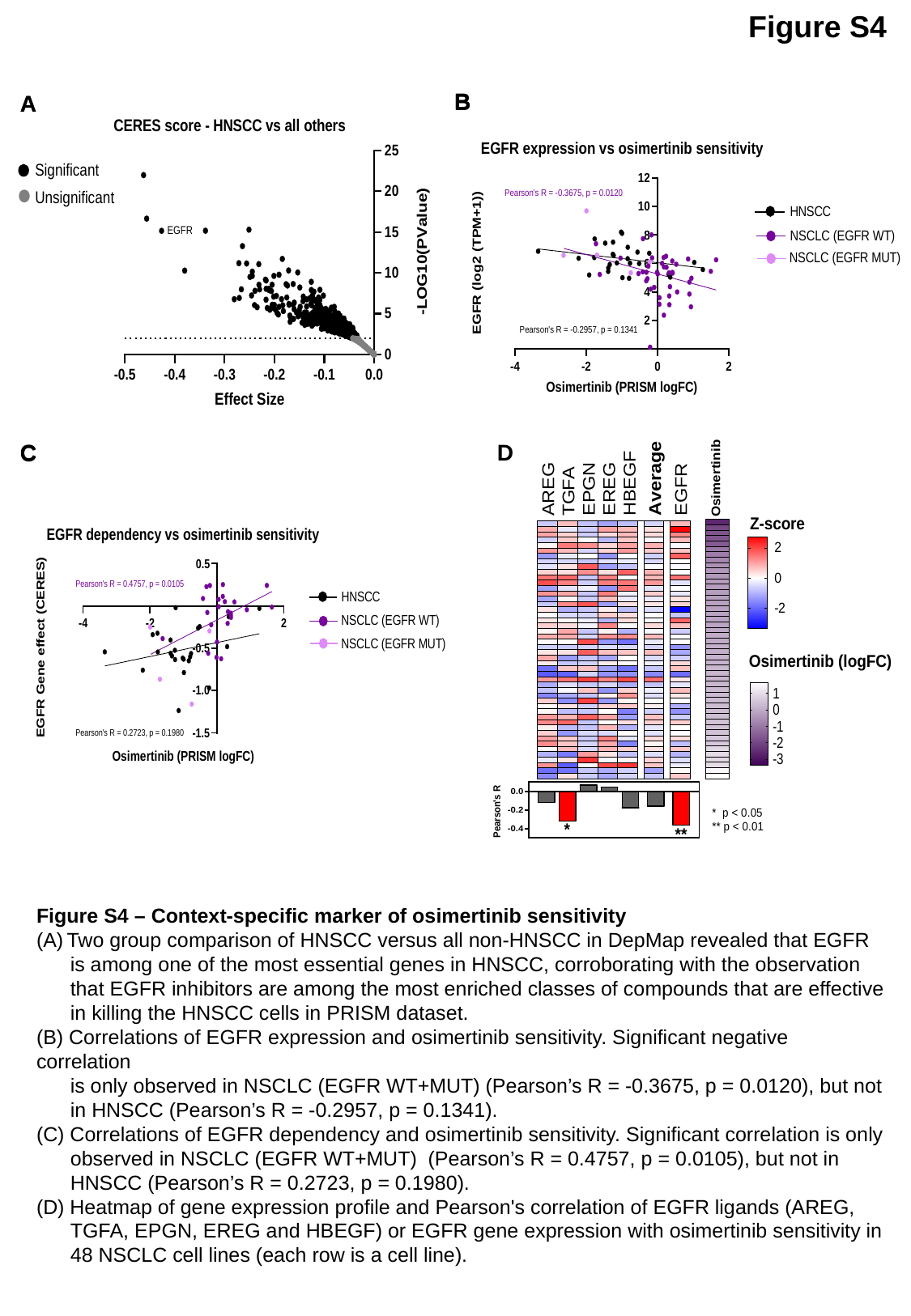

Figure S4
B
B
A
A
D
C
C
Figure S4 – Context-specific marker of osimertinib sensitivity
Two group comparison of HNSCC versus all non-HNSCC in DepMap revealed that EGFR
 is among one of the most essential genes in HNSCC, corroborating with the observation
 that EGFR inhibitors are among the most enriched classes of compounds that are effective
 in killing the HNSCC cells in PRISM dataset.
(B) Correlations of EGFR expression and osimertinib sensitivity. Significant negative correlation
 is only observed in NSCLC (EGFR WT+MUT) (Pearson’s R = -0.3675, p = 0.0120), but not
 in HNSCC (Pearson’s R = -0.2957, p = 0.1341).
(C) Correlations of EGFR dependency and osimertinib sensitivity. Significant correlation is only
 observed in NSCLC (EGFR WT+MUT) (Pearson’s R = 0.4757, p = 0.0105), but not in
 HNSCC (Pearson’s R = 0.2723, p = 0.1980).
(D) Heatmap of gene expression profile and Pearson's correlation of EGFR ligands (AREG,
 TGFA, EPGN, EREG and HBEGF) or EGFR gene expression with osimertinib sensitivity in
 48 NSCLC cell lines (each row is a cell line).

## Slide 6
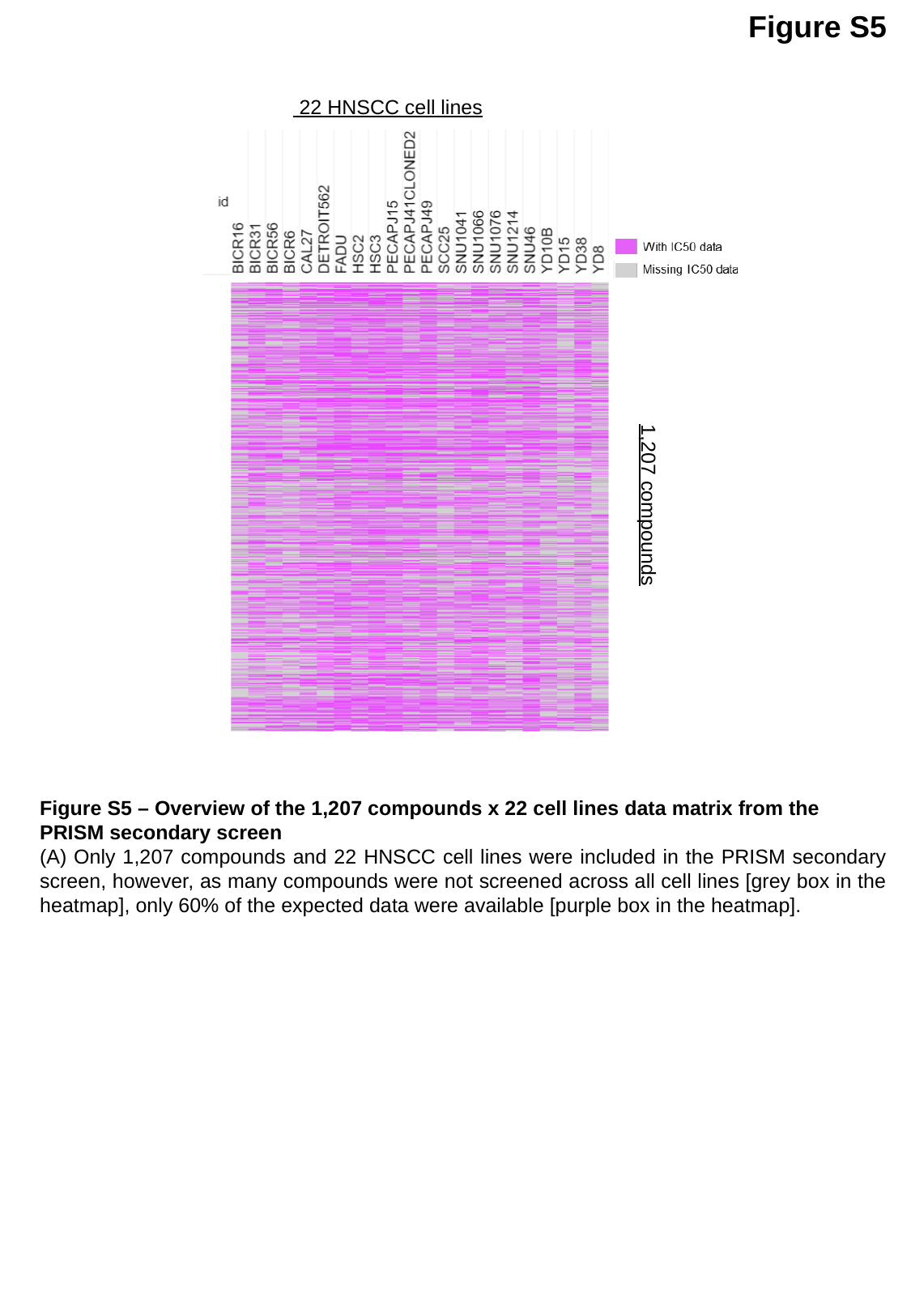

Figure S5
 22 HNSCC cell lines
1,207 compounds
Figure S5 – Overview of the 1,207 compounds x 22 cell lines data matrix from the PRISM secondary screen
(A) Only 1,207 compounds and 22 HNSCC cell lines were included in the PRISM secondary screen, however, as many compounds were not screened across all cell lines [grey box in the heatmap], only 60% of the expected data were available [purple box in the heatmap].
